# Real-world safety of vericiguat as a fifth-line agent in heart failure: a pharmacovigilance study of quintuple therapy

**DOI:** 10.3389/fcvm.2026.1854050

**Published:** 2026-10-13

**Authors:** Daqiu Chen, Yanqing Wu, Zixun Wang, Zhanxiong Xie, Shanghua Xu, Shunxiang Luo

**Affiliations:** 1 Department of Cardiology, Nanping First Hospital Affiliated to Fujian Medical University, Nanping, Fujian, China; 2 Peking University Institute of Cardiovascular Sciences, Beijing, China

**Keywords:** anemia, FAERS, guideline-directed medical therapy, heart failure, pharmacovigilance, SGLT2 inhibitors, vericiguat

## Abstract

**Background:**

Vericiguat, a novel soluble guanylate cyclase (sGC) stimulator, is indicated for high-risk worsening heart failure (HF). However, its real-world reporting profile when added to contemporary quadruple guideline-directed medical therapy (GDMT)—effectively creating a “quintuple” regimen—remains largely unexplored, particularly regarding cardiorenal and hematologic adverse-event reporting patterns.

**Methods:**

We conducted a retrospective pharmacovigilance study using the FDA Adverse Event Reporting System (FAERS) database (2021 Q1–2025 Q4). HF reports were strictly stratified into an expanded quadruple GDMT cohort [renin–angiotensin system inhibitor + beta-blocker + mineralocorticoid receptor antagonist [MRA] + sodium–glucose cotransporter-2 inhibitor [SGLT2i]] and a quintuple cohort (GDMT + vericiguat). Disproportionality analysis utilized a dual-algorithm approach: the frequentist Reporting Odds Ratio (ROR) and the Bayesian Information Component (IC) derived from the Bayesian Confidence Propagation Neural Network (BCPNN). SGLT2i-stratified analyses and formal interaction testing were also performed.

**Results:**

The final cohort comprised 5,524 reports (5,459 quadruple; 65 quintuple). No ROR signals were identified for hypotension (ROR = 1.66, 95% CI: 0.90–3.06), syncope (ROR = 0.85, 95% CI: 0.21–3.49), or acute kidney injury (ROR = 0.61, 95% CI: 0.26–1.41). Anemia met the frequentist ROR signal criterion (ROR = 2.61, 95% CI: 1.03–6.58), but not the BCPNN criterion (IC = 0.96, IC−2SD = −0.29). The anemia ROR was 2.61 (95% CI: 1.03–6.58) with an SGLT2i background and 1.82 (95% CI: 0.42–7.88) without an SGLT2i background; formal interaction testing did not demonstrate significant effect modification (*P* for interaction = 0.683). No statistically significant differences were detected in reported serious outcomes between groups.

**Conclusions:**

In this FAERS analysis, no robust reporting signals were identified for hypotension, syncope, or acute kidney injury after vericiguat was added to contemporary quadruple GDMT. Although anemia met the frequentist ROR criterion, the finding was not supported by Bayesian signal detection, and formal analyses did not demonstrate significant effect modification by background SGLT2i use. Given the small vericiguat-exposed cohort, these findings should be considered hypothesis-generating and require confirmation in larger longitudinal studies.

## 1 Introduction

Heart failure (HF) is a global public health issue with a high incidence and mortality rate, and it represents a significant burden on healthcare systems (1). In recent years, the treatment strategy for heart failure with reduced ejection fraction (HFrEF) has undergone a shift. Contemporary guidelines recommend four foundational pharmacological classes for HFrEF: renin-angiotensin system inhibitors [RASi; including angiotensin receptor–neprilysin inhibitors (ARNI), angiotensin-converting enzyme inhibitors (ACEI), or angiotensin receptor blockers (ARB)], beta-blockers, mineralocorticoid receptor antagonists (MRAs), and sodium-glucose cotransporter-2 inhibitors (SGLT2i) (2). Together, these therapies substantially reduce the risks of cardiovascular death and heart failure hospitalization.

Despite the significant clinical benefits of quadruple therapy, cardiovascular death and hospitalization risks remain high in patients with worsening or advanced heart failure (3, 4). Vericiguat, an oral soluble guanylate cyclase (sGC) stimulator, enhances the nitric oxide (NO)–sGC–cyclic guanosine monophosphate (cGMP) pathway, which is impaired in heart failure (5). In the VICTORIA trial (Vericiguat in Patients with Heart Failure and Reduced Ejection Fraction), adding vericiguat to standard treatment significantly reduced the risk of the composite outcome of cardiovascular death or first heart failure hospitalization (6). Accordingly, vericiguat is recommended as an additional therapeutic option for selected patients with worsening HFrEF who remain at high risk despite guideline-directed medical therapy (GDMT) (7).

Recent real-world studies have described vericiguat use in patients receiving highly optimized contemporary GDMT, including frequent concomitant use of SGLT2 inhibitors (8–10). However, post-marketing adverse-event reporting patterns associated with concomitant vericiguat and SGLT2i therapy remain incompletely characterized. This issue is clinically relevant because vericiguat has vasodilatory effects, whereas SGLT2 inhibitors influence volume status, renal hemodynamics, and erythropoiesis. Moreover, anemia is a common and prognostically significant comorbidity in heart failure patients, affecting approximately 20%–30% of this population (11, 12). SGLT2 inhibitors have been shown to increase hemoglobin and hematocrit levels in patients with HF (13), whereas anemia has been reported more frequently with vericiguat than with placebo in the VICTORIA trial (14). However, pharmacovigilance evidence specifically characterizing anemia reporting with concomitant vericiguat and SGLT2i therapy in contemporary multidrug HF treatment remains limited. These considerations support post-marketing evaluation of hemodynamic, renal, and hematologic adverse-event reporting patterns in patients receiving increasingly intensive multidrug therapy.

This study used the FDA Adverse Event Reporting System (FAERS) database to characterize adverse-event reporting patterns associated with the addition of vericiguat to contemporary expanded quadruple GDMT. Specifically, we sought to evaluate pre-specified reporting signals for hypotension, syncope, acute kidney injury, and anemia; conduct an exploratory phenome-wide reporting scan; describe time-to-onset and reported serious outcomes; and explore whether reporting estimates differed according to background SGLT2i use. Given the inherent limitations of spontaneous reporting data, the study was designed for signal detection and hypothesis generation rather than estimation of absolute adverse-event incidence or causal drug effects.

## 2 Materials and methods

### 2.1 Data source and preprocessing

This retrospective pharmacovigilance study utilized data from the US Food and Drug Administration Adverse Event Reporting System (FAERS) (15). The study period was defined from the first quarter of 2021 (2021 Q1) to the fourth quarter of 2025 (2025 Q4) to capture contemporary post-marketing use of vericiguat within the evolving landscape of guideline-directed medical therapy for heart failure. Raw FAERS data, including demographic information, drug information, adverse event preferred terms (PTs) coded using the Medical Dictionary for Regulatory Activities (MedDRA) (16), and reported clinical outcomes, were extracted and standardized. Deduplication was performed according to FDA recommendations, retaining the most recent report for each unique case based on PRIMARYID, CASEID, and FDA_DT.

### 2.2 Cohort definition and study population

A multi-step filtration protocol was used to construct treatment cohorts among FAERS reports involving heart failure therapies. The foundational renin–angiotensin system (RAS) inhibitor category included an angiotensin receptor–neprilysin inhibitor (ARNI), angiotensin-converting enzyme inhibitor (ACEI), or angiotensin II receptor blocker (ARB). Reports indicating heart failure were subsequently screened according to the predefined treatment regimens.

The expanded quadruple therapy group comprised reports involving a RAS inhibitor, beta-blocker, mineralocorticoid receptor antagonist (MRA), and sodium-glucose cotransporter-2 inhibitor (SGLT2i). The quintuple therapy group comprised reports involving vericiguat in addition to all four components of expanded quadruple therapy.

No exclusions based specifically on pre-existing severe anemia or end-stage renal disease were applied because FAERS does not provide sufficiently complete longitudinal clinical information to consistently distinguish pre-existing from incident conditions.

### 2.3 Pharmacovigilance and disproportionality analysis

Disproportionality analyses were performed to characterize adverse-event reporting patterns associated with the addition of vericiguat. Four adverse events were pre-specified: hypotension, syncope, anemia, and acute kidney injury. In addition, an exploratory phenome-wide reporting scan across eligible MedDRA PTs was performed and visualized using a volcano plot. Wald *P* values were adjusted for multiple comparisons using the Benjamini–Hochberg false discovery rate (FDR) procedure (17).

Given the limitations of spontaneous reporting data and the small number of vericiguat-exposed reports, complementary frequentist and Bayesian disproportionality approaches were used.

1. Frequentist analysis (ROR) (18): Reporting odds ratios (RORs) and 95% confidence intervals (CIs) were calculated using report-level 2 × 2 contingency tables. The Haldane–Anscombe correction, adding 0.5 to all four cells, was applied only when at least one cell contained zero observations. An ROR signal was defined as a lower 95% confidence limit greater than 1.0. Detailed mathematical formulations are provided in the Supplementary Methods.
2. Bayesian shrinkage analysis (BCPNN) (19): The Bayesian Confidence Propagation Neural Network (BCPNN) method was used as a complementary approach to calculate the Information Component (IC) and its uncertainty. A Bayesian signal was defined as an IC lower bound (IC−2SD) greater than 0. Detailed mathematical formulations are provided in the Supplementary Methods.

### 2.4 Time-to-onset and reported serious outcomes

Time-to-onset (TTO) was evaluated among reports with evaluable index and event dates. Drug records matching the predefined TTO target-drug dictionary were linked to the FAERS THER table, and the latest available THER start date within each report was used as the index date. The reported event date was obtained from the DEMO table, and TTO was calculated as the interval, in days, between the index date and the event date. Given the limited availability of evaluable dates, TTO analyses were considered descriptive and exploratory. Empirical cumulative distribution functions (ECDFs) were used to visualize TTO, with Figure 3 restricted to the first 365 days. The 365-day restriction was applied for visualization only and was not used to calculate the summary TTO statistics.

Reported serious outcomes were categorized as death, life-threatening events, hospitalization, and other serious outcomes and were compared between treatment groups. These outcome categories were not mutually exclusive, and a single FAERS report could contribute to more than one category.

### 2.5 Sensitivity analysis according to background SGLT2 inhibitor use

To explore whether adverse-event reporting estimates differed according to background SGLT2 inhibitor use, sensitivity analyses were performed within SGLT2i-containing and SGLT2i-free treatment backgrounds. In the SGLT2i-containing analysis, quintuple therapy was compared with expanded quadruple therapy. In the SGLT2i-free analysis, triple therapy plus vericiguat was compared with triple therapy alone, where triple therapy comprised a RAS inhibitor, beta-blocker, and MRA.

Stratified RORs and 95% CIs were estimated for the pre-specified adverse events. For anemia, formal effect-modification analyses were additionally performed using logistic regression with a vericiguat × SGLT2i interaction term. Model 0 was unadjusted, whereas Model 1 adjusted for age, sex, reporting year, reporter type, and country.

### 2.6 Vericiguat dose-data assessment

Available vericiguat dose information was descriptively assessed among reports in the quintuple therapy group. Dose-response modeling was not performed because clinically interpretable dose strata and adverse-event counts within individual dose categories were too sparse to support reliable estimation.

### 2.7 Statistical analysis

All data processing and statistical analyses were performed using R software (version 4.4.1). Continuous variables were summarized as medians with interquartile ranges (IQRs), and categorical variables as counts and percentages. Between-group comparisons were performed using the Mann–Whitney U test for continuous variables and Pearson's chi-square test or Fisher's exact test for categorical variables, as appropriate. Reported serious outcomes were compared using Fisher's exact test.

For the exploratory phenome-wide reporting scan, multiple testing was controlled using the Benjamini–Hochberg FDR procedure. Logistic regression was used for formal vericiguat × SGLT2i interaction analyses as described above. Conventional hypothesis tests were two-sided, with *P* < 0.05 considered statistically significant. Disproportionality signals were evaluated according to the pre-specified ROR and BCPNN criteria rather than conventional *P*-value thresholds.

## 3 Results

### 3.1 Population characteristics and cohort selection

From the initial 11,842,356 reports in the FAERS database (2021 Q1–2025 Q4), a multi-step filtration process was employed (Figure 1). By expanding the baseline definition of GDMT to include a broader range of renin-angiotensin system inhibitors (ARNI, ACEI, or ARB) alongside beta-blockers, MRAs, and SGLT2 inhibitors, the predefined heart failure treatment cohorts were constructed. The final study population comprised 5,524 reports, including 5,459 reports receiving expanded quadruple therapy and 65 reports receiving quintuple therapy with vericiguat.

**Figure 1:**
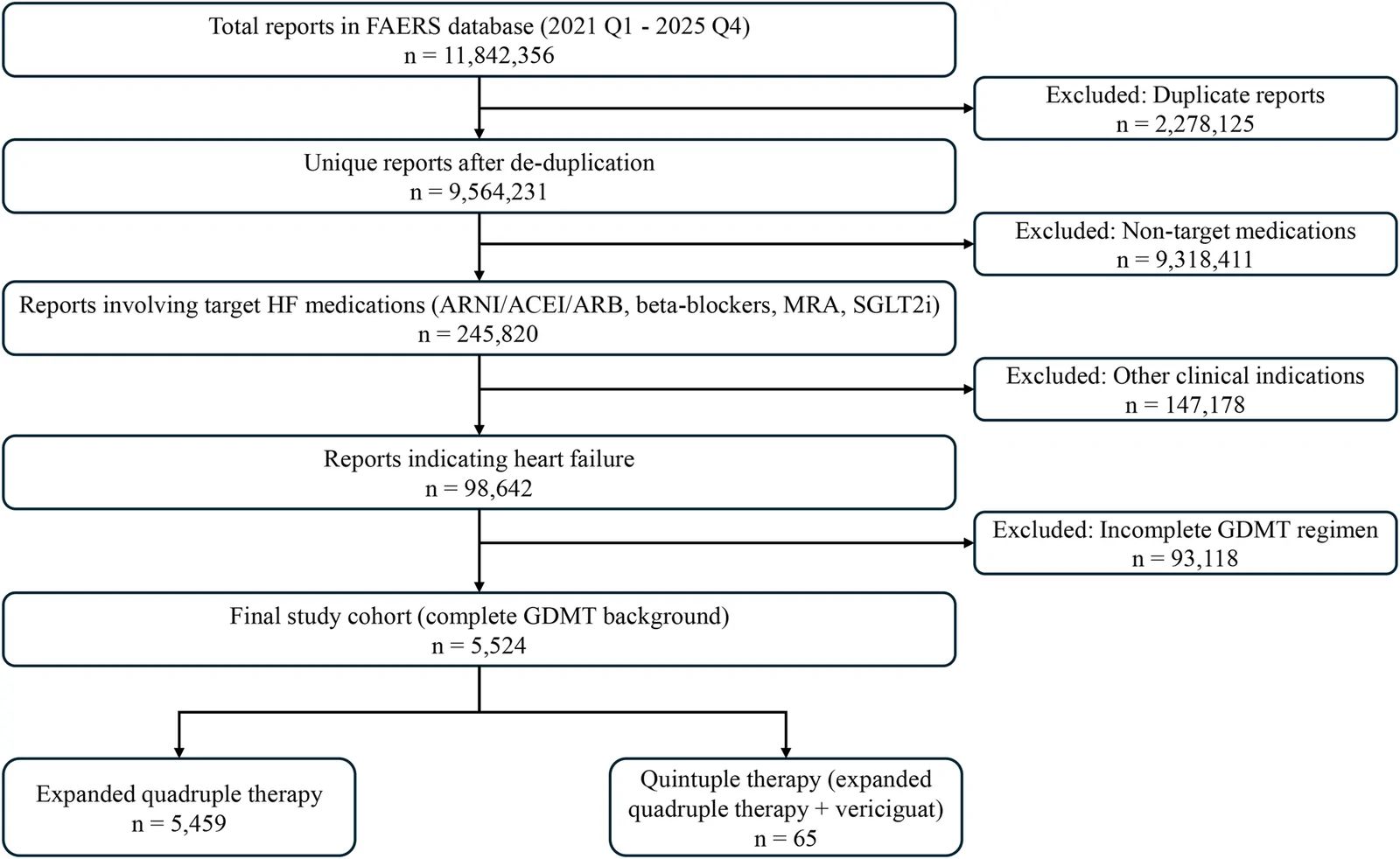
Flowchart of report selection and cohort construction. FAERS reports from 2021 Q1 to 2025 Q4 were deduplicated and screened for heart failure medication exposure, heart failure indication, and complete GDMT background. The final cohort included 5,459 reports receiving expanded quadruple therapy and 65 receiving quintuple therapy with vericiguat. ACEI, angiotensin-converting enzyme inhibitor; ARB, angiotensin receptor blocker; ARNI, angiotensin receptor–neprilysin inhibitor; FAERS, FDA Adverse Event Reporting System; GDMT, guideline-directed medical therapy; MRA, mineralocorticoid receptor antagonist; SGLT2i, sodium–glucose cotransporter-2 inhibitor.

Demographic and reporting characteristics are summarized in Table 1. The median age was 70.0 years in both the quintuple (IQR: 60.8–80.8) and expanded quadruple groups (IQR: 61.0–78.0; *P* = 0.542). There were no significant differences regarding sex distribution (male: 61.5% vs. 58.8%, *P* = 0.461) or body weight (72.4 kg vs. 80.0 kg, *P* = 0.282). Reporter type differed between groups, with healthcare professionals accounting for 75.4% of quintuple-therapy reports and 56.7% of expanded-quadruple-therapy reports (*P* = 0.002). Reporting year also differed between groups (*P* < 0.001).

**Table 1:** Demographic and reporting characteristics of heart failure reports receiving quintuple versus expanded quadruple therapy.

Characteristic	Expanded quadruple therapy (*n* = 5,459)	Quintuple therapy (*n* = 65)	*P* value
Age (years), median [IQR]	70.0 [61.0–78.0]	70.0 [60.8–80.8]	0.542
Sex, *n* (%)			0.461
Female	1,651 (30.2%)	21 (32.3%)	
Male	3,208 (58.8%)	40 (61.5%)	
Unknown/Missing	600 (11.0%)	4 (6.2%)	
Weight (kg), median [IQR]	80.0 [65.8–97.1]	72.4 [59.4–99.1]	0.282
Reporter type, *n* (%)			0.002
Consumer/Patient	1,173 (21.5%)	3 (4.6%)	
Healthcare Professional	3,094 (56.7%)	49 (75.4%)	
Other/Unknown	1,192 (21.8%)	13 (20.0%)	
Country, *n* (%)			0.859
Non-US	3,652 (66.9%)	44 (67.7%)	
United States	1,371 (25.1%)	17 (26.2%)	
Unknown	436 (8.0%)	4 (6.2%)	
Reporting year, *n* (%)			<0.001
2021	380 (7.0%)	0 (0.0%)	
2022	749 (13.7%)	21 (32.3%)	
2023	1,153 (21.1%)	11 (16.9%)	
2024	1,480 (27.1%)	15 (23.1%)	
2025	1,697 (31.1%)	18 (27.7%)	

Continuous variables are presented as median [interquartile range] and categorical variables as *n* (%). Continuous variables were summarized among reports with available values. *P* values were calculated using the Mann–Whitney U test for continuous variables and the Pearson chi-square test or Fisher's exact test for categorical variables, as appropriate. IQR, interquartile range.

### 3.2 Disproportionality and pharmacovigilance analysis

The adverse-event reporting profile associated with adding vericiguat to expanded quadruple therapy was evaluated using complementary frequentist and Bayesian disproportionality analyses (Table 2). An exploratory phenome-wide reporting scan across eligible MedDRA preferred terms was visualized using a volcano plot (Figure 2). Among the four pre-specified adverse events, anemia was the only event meeting the frequentist ROR signal criterion. Hypotension, syncope, and acute kidney injury did not meet this criterion. Several background preferred terms reached false discovery rate significance in the exploratory scan, but these findings were considered exploratory.

**Table 2:** Reporting disproportionality of prespecified adverse events in quintuple versus expanded quadruple therapy.

Adverse event	Expanded quadruple therapy (*n* = 5,459), *n* (%)	Quintuple therapy (*n* = 65), *n* (%)	ROR (95% CI)	BCPNN IC (IC−2SD to IC + 2SD)	ROR signal	BCPNN signal
Hypotension	715 (13.1%)	13 (20.0%)	1.66 (0.90–3.06)	0.53 (−0.32 to 1.38)	No	No
Syncope	197 (3.6%)	2 (3.1%)	0.85 (0.21–3.49)	−0.18 (−1.89 to 1.54)	No	No
Anemia	169 (3.1%)	5 (7.7%)	2.61 (1.03–6.58)	0.96 (−0.29 to 2.21)	Yes	No
Acute kidney injury	783 (14.3%)	6 (9.2%)	0.61 (0.26–1.41)	−0.58 (−1.73 to 0.57)	No	No

Reporting odds ratios were calculated from 2 × 2 contingency tables. The Haldane–Anscombe correction was applied only when at least one cell contained zero observations. An ROR signal was defined as a lower 95% confidence limit >1. Bayesian signal detection was based on the BCPNN method. Reported IC values represent posterior expected IC estimates. A positive Bayesian signal was defined as an IC lower bound (IC−2SD) >0. BCPNN, Bayesian Confidence Propagation Neural Network; CI, confidence interval; IC, information component; ROR, reporting odds ratio.

**Figure 2:**
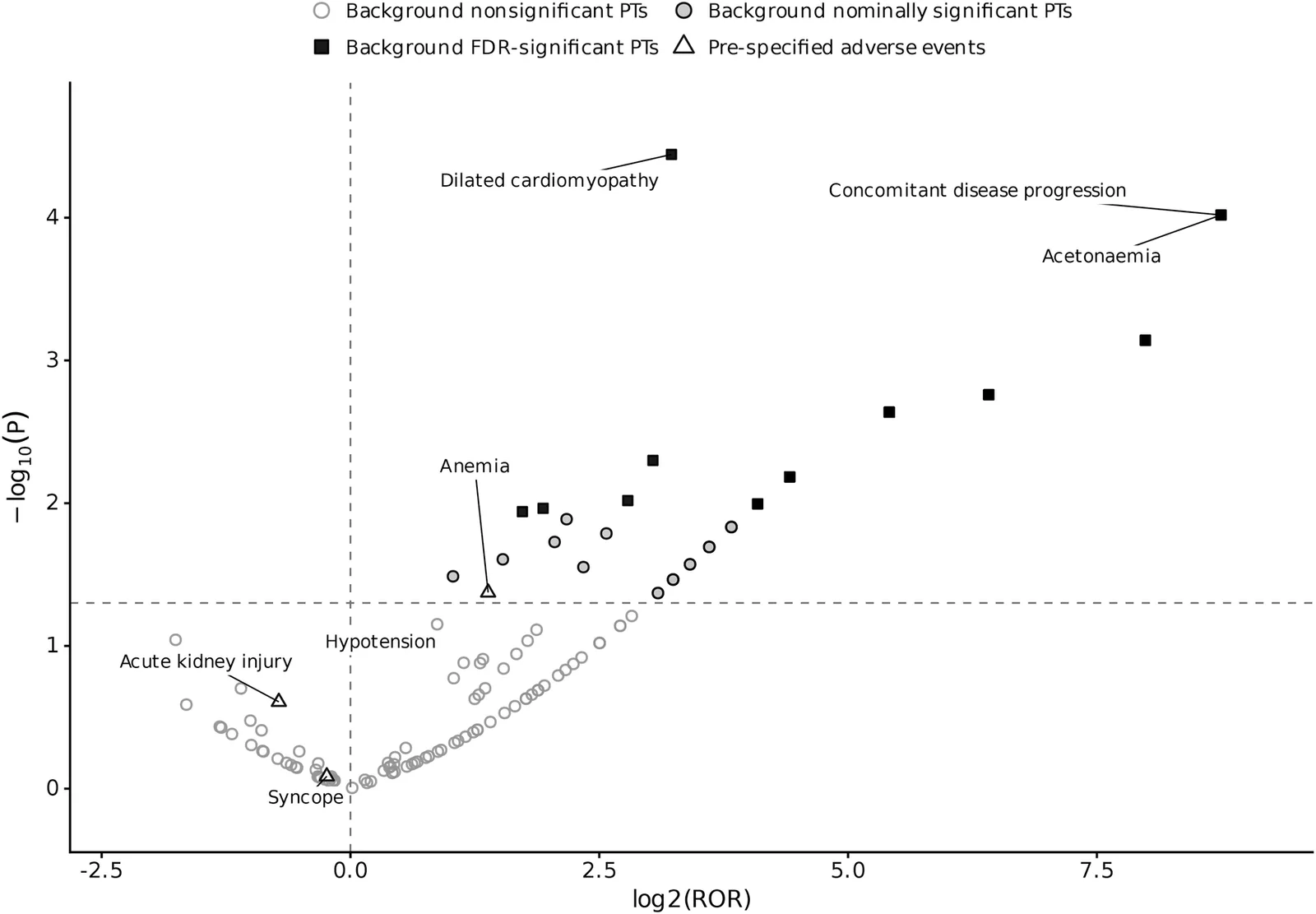
Phenome-wide reporting disproportionality analysis. The vertical dashed line indicates log2(ROR) = 0 (ROR = 1), and the horizontal dashed line indicates *P* = 0.05. Triangles denote the four pre-specified adverse events, and black squares indicate background preferred terms significant after false discovery rate correction. FDR, false discovery rate; PT, preferred term; ROR, reporting odds ratio.

For anemia, the ROR was 2.61 (95% CI: 1.03–6.58). However, the corresponding BCPNN estimate was IC = 0.96 (IC−2SD to IC + 2SD: −0.29 to 2.21), and therefore anemia did not meet the predefined Bayesian signal criterion. Thus, anemia met the frequentist ROR criterion but not the BCPNN criterion.

No ROR signals were identified for hypotension (ROR = 1.66, 95% CI: 0.90–3.06), syncope (ROR = 0.85, 95% CI: 0.21–3.49), or acute kidney injury (ROR = 0.61, 95% CI: 0.26–1.41).

### 3.3 Time-to-onset analysis

The temporal distribution of reported adverse events was evaluated among reports with evaluable index and event dates and was visualized using empirical cumulative distribution functions (ECDFs) (Figure 3A). Evaluable TTO data were available for 809 of 5,459 reports in the expanded quadruple group and 16 of 65 reports in the quintuple group.

**Figure 3:**
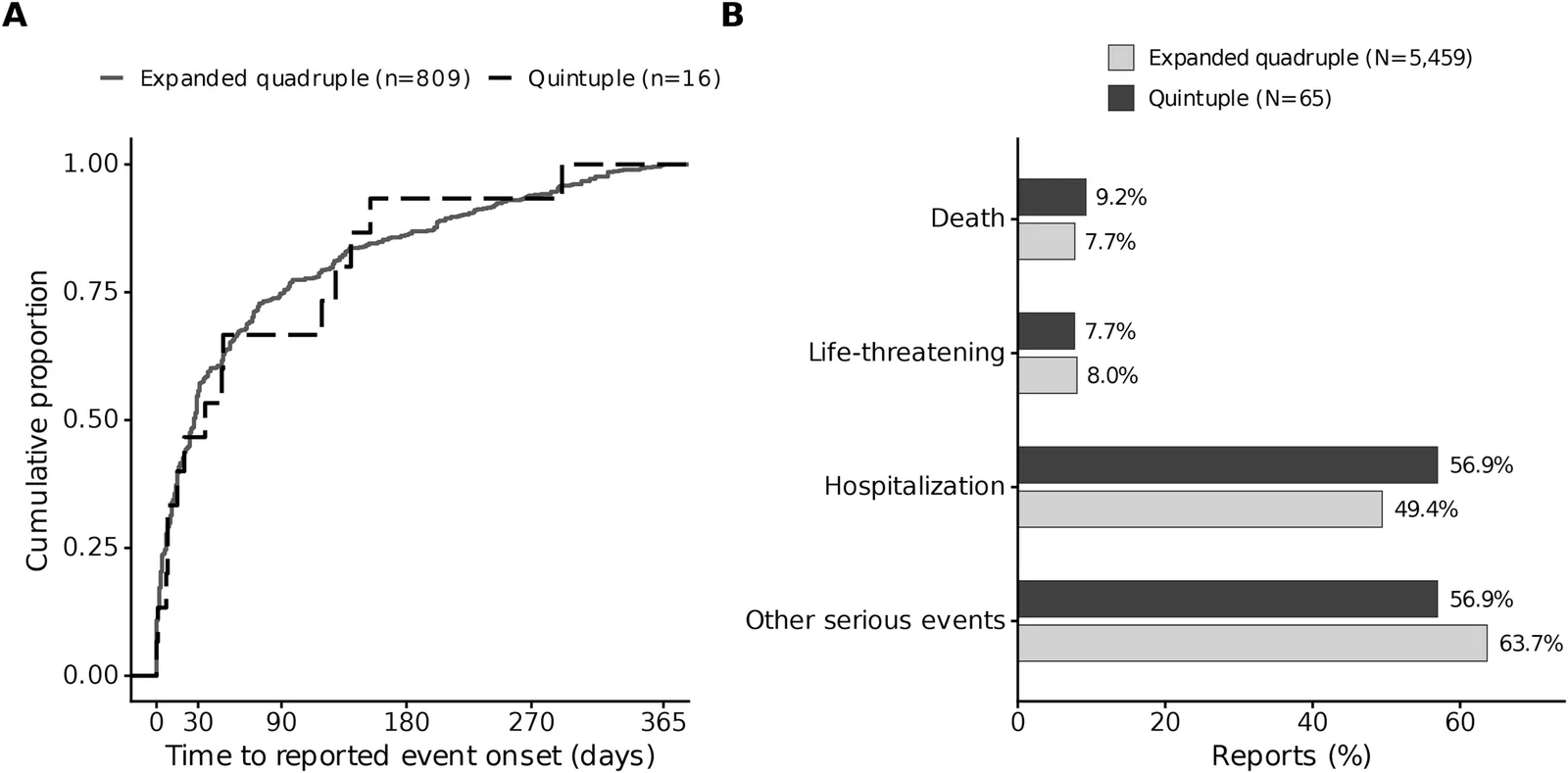
Time-to-onset distribution and reported serious outcomes. **(A)** Empirical cumulative distribution of TTO among reports with evaluable index and event dates (expanded quadruple therapy, *n* = 809; quintuple therapy, *n* = 16), displayed through 365 days. **(B)** Proportions of reported serious outcomes in the expanded quadruple therapy (*N* = 5,459) and quintuple therapy (*N* = 65) groups. Serious outcome categories were not mutually exclusive.

The median TTO was 39 days (IQR: 10–221) in the expanded quadruple group and 41 days (IQR: 8–132) in the quintuple group. Given the limited number of evaluable reports, particularly in the quintuple group, these findings were considered descriptive and exploratory.

### 3.4 Reported serious outcomes

Reported serious outcomes were compared between the quintuple and expanded quadruple therapy groups (Table 3 and Figure 3B). No statistically significant differences were detected for death (9.2% vs. 7.7%, *P* = 0.638), life-threatening events (7.7% vs. 8.0%, *P* = 1.000), hospitalization (56.9% vs. 49.4%, *P* = 0.262), or other serious events (56.9% vs. 63.7%, *P* = 0.300).

**Table 3:** Reported serious outcomes in quintuple versus expanded quadruple therapy.

Reported serious outcome	Expanded quadruple therapy (*n* = 5,459), *n* (%)	Quintuple therapy (*n* = 65), *n* (%)	*P* value
Death	422 (7.7%)	6 (9.2%)	0.638
Life-threatening	438 (8.0%)	5 (7.7%)	1.000
Hospitalization	2,698 (49.4%)	37 (56.9%)	0.262
Other serious events	3,475 (63.7%)	37 (56.9%)	0.300

Data are presented as *n* (%). Outcome categories were not mutually exclusive; a single FAERS report could contribute to more than one serious outcome category. *P* values were calculated using Fisher's exact test. FAERS, FDA Adverse Event Reporting System.

### 3.5 Sensitivity analysis according to background SGLT2 inhibitor use

To further explore whether adverse-event reporting estimates differed according to background SGLT2 inhibitor use, sensitivity analyses were performed using an SGLT2i-containing background (quintuple vs. expanded quadruple therapy) and an SGLT2i-free background (triple therapy plus vericiguat vs. triple therapy alone). The SGLT2i-free comparison included 19 reports receiving triple therapy plus vericiguat and 13,613 reports receiving triple therapy alone.

For anemia, the ROR was 2.61 (95% CI: 1.03–6.58) in the SGLT2i-containing stratum and 1.82 (95% CI: 0.42–7.88) in the SGLT2i-free stratum (Figure 4). Although the point estimate was numerically higher in the SGLT2i-containing stratum, formal interaction testing did not demonstrate statistically significant effect modification (unadjusted Model 0, *P* for interaction = 0.683; adjusted Model 1, *P* for interaction = 0.888).

**Figure 4:**
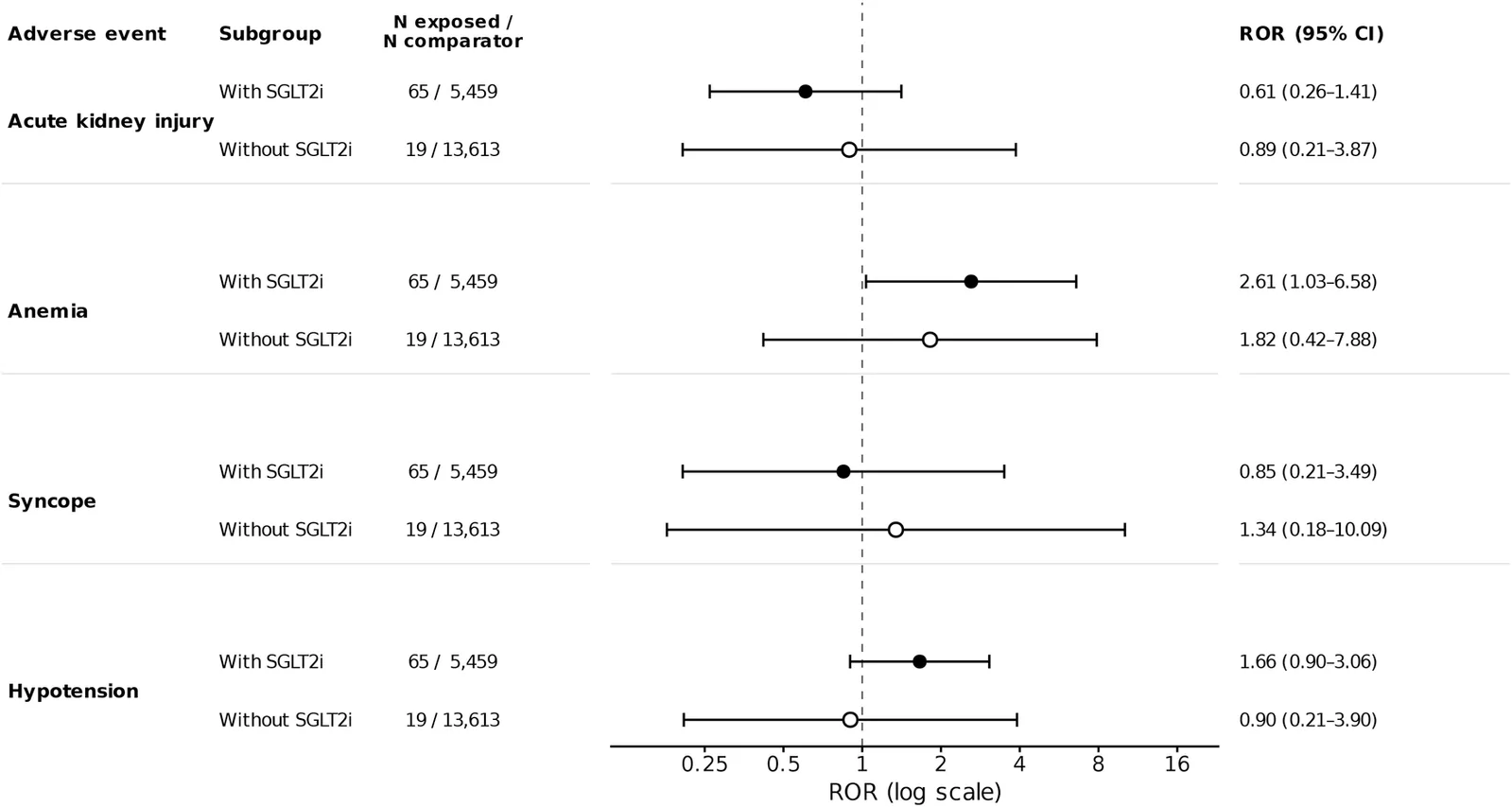
Reporting odds ratios stratified by background SGLT2 inhibitor use. RORs and 95% CIs are shown for pre-specified adverse events with and without background SGLT2 inhibitor therapy. The vertical dashed line indicates ROR = 1. For anemia, formal interaction testing was not significant (*P* for interactio*n* = 0.683). CI, confidence interval; ROR, reporting odds ratio; SGLT2i, sodium–glucose cotransporter-2 inhibitor.

No ROR signals for hypotension, syncope, or acute kidney injury were identified in either SGLT2i stratum.

### 3.6 Vericiguat dose-data availability

Interpretable vericiguat dose information was available for 42 of 65 quintuple-therapy reports (64.6%). However, the numbers of reports and adverse events within individual dose strata were too sparse to support reliable dose-response estimation. Therefore, no formal dose-response analysis was performed.

## 4 Discussion

This study evaluated the post-marketing adverse-event reporting profile of vericiguat when added to contemporary expanded quadruple guideline-directed medical therapy (GDMT) using the FAERS database. Several findings warrant attention. First, no ROR signals were identified for hypotension, syncope, or acute kidney injury (AKI). Second, anemia met the frequentist ROR signal criterion but did not meet the Bayesian Confidence Propagation Neural Network (BCPNN) criterion. Third, although the anemia ROR was numerically higher in the SGLT2i-containing stratum, formal interaction testing did not demonstrate statistically significant effect modification. Finally, no statistically significant differences were detected in reported serious outcomes between treatment groups, whereas the time-to-onset (TTO) analysis remained exploratory because evaluable index and event dates were available for only a small proportion of reports.

Vericiguat, a soluble guanylate cyclase (sGC) stimulator, enhances the impaired nitric oxide–sGC–cyclic guanosine monophosphate pathway and may exert vasodilatory effects (5). Because contemporary HF treatment frequently combines multiple agents with hemodynamic and renal effects, hypotension, syncope, and renal adverse events are clinically relevant considerations when vericiguat is added to background GDMT. In the present analysis, no disproportionate reporting signals were identified for hypotension, syncope, or AKI. These observations are broadly consistent with randomized trial data showing no marked excess of symptomatic hypotension or syncope with vericiguat and with analyses from VICTORIA indicating that the treatment effect of vericiguat was generally preserved across a wide range of renal function (20, 21). However, the absence of a disproportionality signal should not be interpreted as evidence of absence of risk, particularly given the small number of vericiguat-exposed reports and the limitations inherent to spontaneous reporting systems.

The anemia finding requires more nuanced interpretation. In the VICTORIA trial, anemia was reported slightly more frequently in the vericiguat group than in the placebo group (7.6% vs. 5.7%) (14). In our FAERS analysis, anemia met the frequentist ROR signal criterion (ROR = 2.61, 95% CI: 1.03–6.58). In contrast, the Bayesian estimate did not meet the predefined BCPNN signal criterion (IC = 0.96; IC−2SD = −0.29). The discordance between the two approaches indicates that the anemia finding was not consistently supported across signal-detection methods and should therefore be interpreted cautiously. Importantly, disproportionality analyses quantify differential reporting rather than absolute incidence and cannot establish that vericiguat causes anemia.

The SGLT2i-stratified analysis further illustrates the need for caution. The anemia ROR was 2.61 (95% CI: 1.03–6.58) in the SGLT2i-containing stratum and 1.82 (95% CI: 0.42–7.88) in the SGLT2i-free stratum. Although the point estimate was numerically higher with an SGLT2i background, formal interaction testing did not show statistically significant effect modification in either the unadjusted model (*P* for interaction = 0.683) or the adjusted model (*P* for interaction = 0.888). Therefore, these data do not support a confirmed pharmacological interaction between vericiguat and SGLT2 inhibitors with respect to anemia reporting.

This finding should also be considered in the context of the known hematologic effects of SGLT2 inhibitors, which generally increase hemoglobin and hematocrit through mechanisms involving erythropoiesis and iron mobilization (22, 23). The numerical difference between strata may instead reflect sparse data, reporting variability, residual differences in disease severity, or other unmeasured characteristics of patients receiving more intensive multidrug therapy. Patients with advanced HF are also susceptible to anemia because of cardiorenal dysfunction, inflammation, and disturbances in iron homeostasis (24, 25). Accordingly, the present findings are more appropriately regarded as hypothesis-generating than as evidence of pharmacological synergy.

The TTO analysis should likewise be interpreted descriptively. Evaluable TTO information was available for 809 reports in the expanded quadruple group but only 16 reports in the quintuple group. Median TTO was 39 days (IQR, 10–221) and 41 days (IQR, 8–132), respectively. Given the limited number of evaluable vericiguat reports, the present data do not support inference of a specific high-risk temporal window for adverse events.

Dose information was also incomplete. Although interpretable vericiguat dose data were available for 42 of 65 reports, individual dose strata and adverse-event counts were too sparse for reliable dose-response estimation. Consequently, these FAERS data do not establish an evidence-based schedule for hematologic monitoring or support dose-specific safety conclusions. Clinical monitoring should therefore continue to follow individual patient characteristics and established HF management practices rather than a monitoring interval derived from the present analysis.

Several limitations should be considered when interpreting these findings. First, FAERS is a spontaneous reporting system and is subject to underreporting, stimulated reporting, differential reporting, missing data, and the absence of a reliable denominator for the total number of exposed patients; therefore, absolute incidence rates and comparative clinical risks cannot be estimated (15). Second, only 65 reports met the quintuple-therapy definition, resulting in wide confidence intervals and limited ability to evaluate rare events or perform extensive subgroup analyses. Third, FAERS lacks sufficiently complete information on HF severity, comorbidities, longitudinal renal function, baseline anemia, iron status, and other potential confounders. Pre-existing severe anemia and end-stage renal disease could therefore not be reliably distinguished from incident conditions. Fourth, longitudinal laboratory measurements, including serial hemoglobin, ferritin, and other hematologic biomarkers, were unavailable. Fifth, TTO data were evaluable in only 16 vericiguat-exposed reports, and dose information was insufficient for reliable dose-response analysis. Finally, although the active treatment comparator and formal interaction analyses were intended to improve interpretability, residual confounding and reporting bias cannot be eliminated, and disproportionality signals should not be interpreted as evidence of causality.

## 5 Conclusions

In this FAERS analysis, no robust reporting signals were identified for hypotension, syncope, or acute kidney injury with vericiguat added to contemporary expanded quadruple GDMT. Anemia met the frequentist ROR criterion but not the BCPNN criterion, and no significant interaction with background SGLT2 inhibitor use was demonstrated. These findings are hypothesis-generating and warrant confirmation in larger longitudinal studies.

## Data Availability

The original contributions presented in the study are included in the article/Supplementary Material, further inquiries can be directed to the corresponding authors.
