# Supplementary material for: Real-world safety of vericiguat as a fifth-line agent in heart failure: a pharmacovigilance study of quintuple therapy
Source: Front Cardiovasc Med. 2026 Oct 13;13:1854050. doi: 10.3389/fcvm.2026.1854050 (PMC13710072; doi:10.3389/fcvm.2026.1854050)
Supplement: Supplementary file 1 [file Table1.docx]

**Supplementary Methods**

**S1. Reporting Odds Ratio**

For each adverse event, a 2 × 2 contingency table was constructed, where a represents the number of reports of the target adverse event in the quintuple-therapy group, b the number of reports of other adverse events in the quintuple-therapy group, c the number of reports of the target adverse event in the expanded quadruple-therapy group, and d the number of reports of other adverse events in the expanded quadruple-therapy group.

$$ROR=\frac{a/b}{c/d}=\frac{ad}{bc}$$

$$SE\left[ \ln\left( ROR \right) \right]=\sqrt{\frac{1}{a}+\frac{1}{b}+\frac{1}{c}+\frac{1}{d}}$$

$$95\% CI=\exp\left[ \ln\left( ROR \right)\pm1.96\times SE\left\{ \ln\left( ROR \right) \right\} \right.\boldsymbol{]}$$

When at least one cell contained zero observations, the Haldane–Anscombe correction was applied by adding 0.5 to all four cells. An ROR signal was defined as a lower 95% confidence limit greater than 1.0.

**S2. Bayesian Confidence Propagation Neural Network**

The Bayesian Confidence Propagation Neural Network (BCPNN) method was used to calculate the Information Component (IC) and its uncertainty. For notational simplicity, let

$$N\equiv a+b+c+d,\quad N_{x}\equiv a+b,\quad N_{y}\equiv a+c$$

where a, b, c, and d are defined as above.

The observed (unshrunk) Information Component was defined as:

$$IC=\log_{2} \left( \frac{aN}{N_{x}N_{y}} \right)$$

The posterior expected Information Component, which was used as the BCPNN IC estimate reported in the main analysis, was calculated as:

$$E\left( IC \right)\equiv\log_{2} \left[ \frac{\left( a+\gamma_{11} \right)\left( N+\alpha\right)\left( N+\beta\right)}{\left( N+\gamma\right)\left( N_{x}+\alpha_{1} \right)\left( N_{y}+\beta_{1} \right)} \right]$$

The variance of the IC was calculated as:

$$V\left( IC \right)\equiv\frac{1}{\left( \ln2 \right)^{2}}\left\{ \frac{N-a+\gamma-\gamma_{11}}{\left( a+\gamma_{11} \right)\left( 1+N+\gamma\right)}+\frac{N-N_{x}+\alpha-\alpha_{1}}{\left( N_{x}+\alpha_{1} \right)\left( 1+N+\alpha\right)}+\frac{N-N_{y}+\beta-\beta_{1}}{\left( N_{y}+\beta_{1} \right)\left( 1+N+\beta\right)} \right\}$$

where

$$\gamma=\gamma_{11}\frac{\left( N+\alpha\right)\left( N+\beta\right)}{\left( N_{x}+\alpha_{1} \right)\left( N_{y}+\beta_{1} \right)}$$

The prior parameters were specified as:

$$\alpha_{1}=\beta_{1}=1,\quad\alpha=\beta=2,\quad\gamma_{11}=1$$

The posterior standard deviation was obtained as $\sqrt{V\left( IC \right)}$, and the lower and upper bounds were calculated as:

$$\text{IC}+2\text{SD}=E\left( IC \right)+2\sqrt{V\left( IC \right)}$$

$$IC-2SD=E\left( IC \right)-2\sqrt{V\left( IC \right)}$$

A Bayesian signal was defined as an IC lower bound (IC−2SD) greater than 0.
